# Kinetics and Thermodynamics of CO Oxidation by (TiO_2_)_6_

**DOI:** 10.3390/molecules26216415

**Published:** 2021-10-24

**Authors:** Navjot Kaur, Neetu Goel, Michael Springborg, Mohammad Molayem

**Affiliations:** 1 Theoretical and Computational Chemistry Group, Department of Chemistry, Centre of Advanced Studies in Chemistry, Panjab University, Chandigarh 160014, India; Navu1989mann@gmail.com; 2 Guru Gobind Singh College For Women, Sector-26, Chandigarh 160014, India; 3 Physical and Theoretical Chemistry, University of Saarland, 66123 Saarbrücken, Germany; m.molayem@mx.uni-saarland.de

**Keywords:** catalytic effects, metal-oxide clusters, temperature effects

## Abstract

Molecular level insights into the mechanism and thermodynamics of CO oxidation by a (TiO${}_{2}$)${}_{6}$ cluster have been obtained through density functional calculations. Thereby, in this study, as an example, two different structural isomers of (TiO${}_{2}$)${}_{6}$ are considered with the purpose of understanding the interplay between local structure and activity for the CO oxidation reaction. Active sites in the two isomeric forms were identified on the basis of global and local reactivity descriptors. For the oxidation of CO to CO${}_{2}$, the study considered both sequential and simultaneous adsorption of CO and O${}_{2}$ on (TiO${}_{2}$)${}_{6}$ cluster through the ER and LH mechanisms, respectively. Three different pathways were obtained for CO oxidation by (TiO${}_{2}$)${}_{6}$ cluster, and the mechanistic route of each pathway were identified by locating the transition-state and intermediate structures. The effect of temperature on the rate of the reaction was investigated within the harmonic approximation. The structure-dependent activity of the cluster was rationalized through reactivity descriptors and analysis of the frontier orbitals.

## 1. Introduction

The oxidation of carbon monoxide to carbon dioxide holds much importance for the abatement of the environmental pollution. Oxides of various transition metals such as those of iron [1,2,3], cobalt [4,5], nickel [6], and titanium [7,8] have been explored as heterogenous catalysts to facilitate this conversion. Among all the transition metal oxides, titanium oxide has attracted much attention owing to its photocatalytic and hydrophilic properties [7,8,9,10,11]. Several advantages such as high photoactivity, good stability, high corrosion resistance and low price offered by TiO${}_{2}$ nanoparticles make them attractive heterogeneous catalysts [12,13,14]. It has been demonstrated that TiO${}_{2}$ plays a decisive role in CO oxidation by metallic clusters anchored on the surface of TiO${}_{2}$ [15,16,17]. Apart from its role as active support, this metal oxide has found applicability in many industrial processes including solar cells, environmental cleanup and photocatalysis [11,13]. In a recent study, TiO${}_{2}$ supported on graphene has been suggested as a candidate for CO${}_{2}$ reduction [18]. The present work explores the suitability of this transition metal oxide for CO oxidation.

In order to design an effective catalyst for any reaction, a deep understanding of the underlying chemical processes is needed. This, in turn, is sensitive to the size, composition and morphology of the active material. In particular, CO oxidation is reported to show strong dependence on size and composition of the catalyst as well as that of the support material [19,20]. For instance, Reddy and Khanna have investigated the reactivity of a bare Fe${}_{2}$O${}_{3}$ cluster for the oxidation of CO to CO${}_{2}$ through composition changes between Fe${}_{2}$O${}_{2}$ and Fe${}_{2}$O${}_{3}$[21]. The reactivity of Au${}_{m}$Ti${}_{n}$O${}_{2n+x}^{+}$ cluster ions with CO has been examined for different *m*, *n*, and *x* including support from mass spectrometry data [22].

Theoretical studies of the catalytic properties of nanoparticles face several challenges. At first, for sufficiently small nanoparticles (clusters), their properties depend strongly and unpredictably on their size and stoichiometry. Second, even a single cluster will in most cases possess a low symmetry implying that different sites will have different catalytic properties. Third, there may be different paths for the catalytic reaction. Taken together, all these issues make it impossible to obtain a complete description of the catalytic properties of nanoparticles for a given reaction. As an alternative, one may utilize that the catalytic reaction is a local phenomenon that depends mainly on the closest vicinity of the sites at which it takes place. Moreover, by studying some few exemplary cases, one may obtain an understanding of the possible variation in the catalytic properties of the material of interest. In fact, such cluster models have already proven to be very useful to obtain understanding of the catalytic activity at the atomic level. It is this latter approach that will be followed here. Therefore, it is emphasized that the results of this study are not expected to provide accurate information on the specific cluster of the study but rather to give general information on the catalytic properties of TiO${}_{2}$ clusters for the CO oxidation reaction. Accordingly, the present study considers the (TiO${}_{2}$)${}_{6}$ cluster to obtain molecular level insights of the catalytic properties of TiO${}_{2}$ clusters for the CO oxidation. Such models can be treated with accuracy through high level theoretical calculations and hence allow us to peep into structure-reactivity relationships of catalytic materials [23,24,25].

Despite the abundance of literature on the use of such clusters for CO oxidation, the identification of active sites in the clusters and a molecular-level understanding of this reaction remains elusive. The oxidation of CO may proceed either through the Eley-Rideal (ER) or the Langmuir-Hinshelwood (LH) mechanism. In the ER case, CO or O${}_{2}$ in the gas phase reacts with the other species that is chemisorbed on the catalyst, so that the role of the catalyst is determined only by its effect on either CO or O${}_{2}$. In contrast, the LH mechanism involves coadsorption of O${}_{2}$ and CO on the catalyst and yields an intermediate in which both the molecules are activated [26]. The two mechanisms are competitive depending on the compositions of the catalyst and the activation of the adsorbates on the catalyst. In addition to the above two, there exists another possibility, i.e., the Mars-van Krevelen (MvK) mechanisms where the pre-adsorbed CO molecule directly reacts with the nearest surface lattice oxygen atom to form CO${}_{2}$ [27]. The current study explores all the mechanistic routes for the oxidation of CO by a (TiO${}_{2}$)${}_{6}$ cluster. In order to obtain a more general insight into the catalytic properties of TiO${}_{2}$ nanoparticles for this reaction, two different structural isomers of the (TiO${}_{2}$)${}_{6}$ clusters are considered.

## 2. Computational Details

The clusters with high Vertical Electron Affinity (VEA) are reported to be more reactive and best suited for applications in catalysis [28]. Here, the cluster size has been selected by analysing global reactivity descriptors like VEA and hardness (η) reported for (TiO${}_{2}$)${}_{n}$ clusters with n=1→10 by Arab et al. [29]. Since η is related to the cluster’s resistance to deformation or polarization of its electron cloud, n=6 is selected for the present study because of its minimum hardness and high VEA that favors its reaction with the nucleophilic CO [29]. The reactivity of the clusters is strongly influenced by the geometrical arrangement of atoms. Their catalytic properties can be quantified through reactivity descriptors that can be either global or local descriptors. Among those are the vertical ionization energy (VIE) and the VEA that were calculated according to [30]

(1) $$\begin{array}{cc} \mathrm{VIE}= & E_{\mathrm{cation}}-E_{\mathrm{neutral}} \end{array}$$

(2) $$\begin{array}{cc} \mathrm{VEA}= & E_{\mathrm{neutral}}-E_{\mathrm{anion}} \end{array}$$

Here, $E_{\mathrm{cation}}$, $E_{\mathrm{neutral}}$, and $E_{\mathrm{anion}}$ are the energies of the cation at optimised neutral geometry, the neutral, and the anion cluster at its optimized neutral geometry, respectively. For calculating VIE and VEA the single point energy of cation and anion has been calculated by adding charge (positive in case of cation and negative in case of anion) to the optimised geometry of the neutral cluster. Using the VIE and VEA values, global reactivity descriptors (GRD) like hardness (η), electrophilicity (χ), chemical potential (μ), and global electrophilicity index (ω) can be evaluated from [31,32,33]

(3) $$\begin{array}{cc} \mu = & -\frac{1}{2}\left[\mathrm{VIE}+\mathrm{VEA}\right] \end{array}$$

(4) $$\begin{array}{cc} \eta = & \left(\mathrm{VIE}-\mathrm{VEA}\right) \end{array}$$

(5) $$\begin{array}{cc} \omega = & \frac{\mu^{2}}{2\eta } \end{array}$$

In addition, the energy gap, E${}_{\mathrm{gap}}$, between the highest occupied and lowest unoccupied can be a useful descriptor. From position-dependent quantities like the charge density and the Fukui functions, atom-decomposed ones can be extracted. This includes the natural bond order (NBO) which shall be discussed in the present work [34]. Moreover, it becomes possible to study the reactivity at the atomic level using the condensed Fukui functions (FF). The condensed forms for atom *k* in a molecule equals [29,33,35]

(6) $$\begin{array}{cc} f_{k}^{+}= & q_{k}\left(N+1\right)-q_{k}\left(N\right)\;\mathrm{for}\;\mathrm{nucleophilic}\;\mathrm{attack} \end{array}$$

(7) $$\begin{array}{cc} f_{k}^{-}= & q_{k}\left(N\right)-q_{k}\left(N-1\right)\;\mathrm{for}\;\mathrm{electrophilic}\;\mathrm{attack} \end{array}$$

where $q_{k}\left(N\right)$, $q_{k}\left(N+1\right)$, and $q_{k}\left(N-1\right)$ are the charges associated with the *k*th atom in a cluster containing *N*, N+1, and N−1 electrons, respectively.

**Figure 1:**
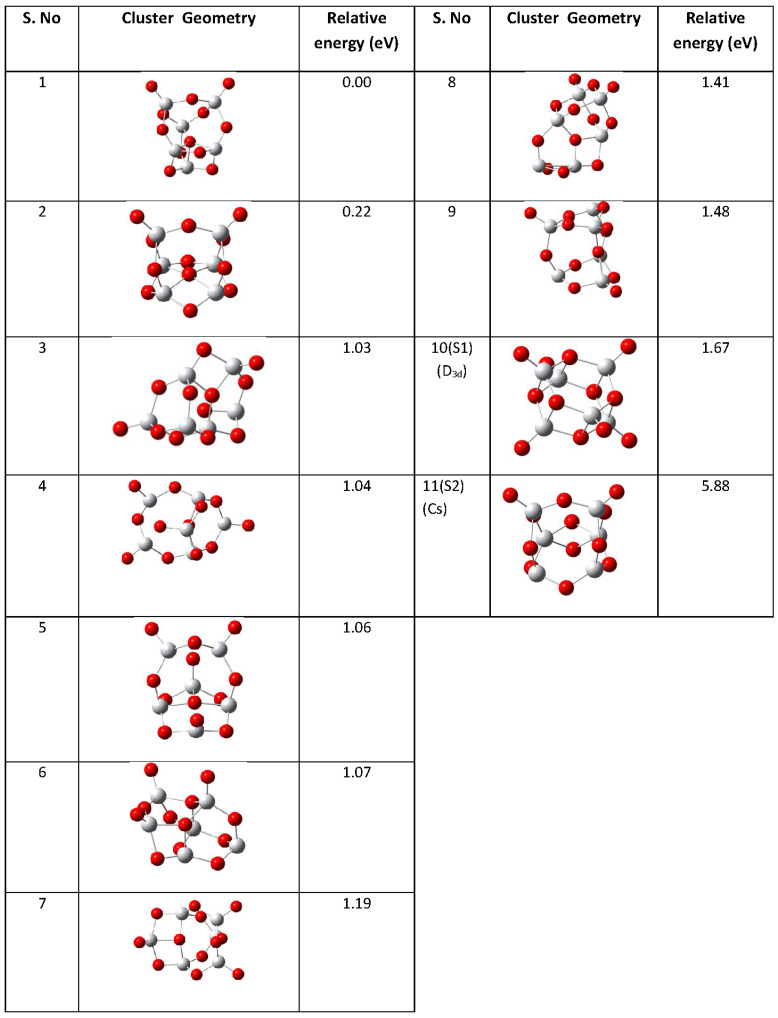
Optimized geometries of isomeric forms of (TiO${}_{2}$)${}_{6}$ cluster in gas phase. Red spheres represent oxygen and grey spheres represent titanium atoms, respectively.

Initially, a set of candidate structures for (TiO${}_{2}$)${}_{6}$ clusters were identified using an approach based on genetic algorithms for the unbiased structure optimization and the DFTB-SCC (Density-Functional Tight-Binding Self-Consistent-Charge) method [36] as implemented in the DFTB+ package [37] for the calculation of the total energy for a given structure. Subsequently, the obtained isomers were re-optimized within density-functional theory using the hybrid B3LYP functional [38,39] in conjunction with the 6-31G(d) [40] basis set for both Ti and O atoms as suggested in the literature [29,41] (shown in Figure 1).

Transition states (TSs) were optimized by using the TS Berny algorithm method [42]. Moreover, vibrational analysis was performed to confirm that intermediates correspond to stable structures and TSs correspond to first order saddle points on the potential energy surface (PES) [43]. Intrinsic reaction coordinate (IRC) calculations [44] established the connectivity of TSs with the local minima. Relative energies of the reactants (R), intermediates (I), TSs, and products (P) were obtained at default temperature and pressure of 298.15 K and 1 atm, respectively.

## 3. Results and Discussion

The relative energies and the geometries of various isomers of (TiO${}_{2}$)${}_{6}$ are shown in Figure 1. The isomers S1 and S2 have been chosen for the study of CO oxidation as they are the most reactive ones. It should be emphasized that the stability of the isomers is only of secondary importance here as the present study is focusing on the catalytic properties of structural elements that can be found in TiO${}_{2}$ nanoparticles.

The S1 isomer has D${}_{3d}$ point group symmetry. Each Ti atom is tetrahedrally coordinated with three bridging oxygen atoms (O${}_{b}$) and one terminal oxygen atom (O${}_{t}$). The S2 isomer has a distorted symmetry and belongs to C${}_{s}$ point group. It has two Ti atoms with three bridging and one terminal oxygen atom (O${}_{t}$) while the remaining four Ti atoms have only bridging oxygen atoms as nearest neighbors.

The reactivity of the cluster species can be quantified in terms of the global and local reactivity descriptors mentioned above. The GRD based descriptors define global reactivity of the cluster as a whole while the LRDs provide estimates of the local selectivity of different sites within the cluster. Since most of these descriptors are obtained as the derivatives of energy and electron density variables, they are reliable parameters in scrutinizing the structure-activity relationship. The values of the GRDs including those of μ, η and ω (obtained using Equations (3)–(5)) for both S1 and S2 isomers of (TiO${}_{2}$)${}_{6}$ cluster in gas phase are given in Table 1. η and μ are key indicators of the overall reactivity of the molecule and the most fundamental descriptors of charge/electron transfer in a chemical reaction. The hardness is related to the cluster’s resistance to a deformation or polarization of its electron cloud. The electronic chemical potential can be regarded as the escaping tendency of an electron from a cluster in its ground state. The larger values of η and E${}_{\mathrm{gap}}$, and lower values of μ for the S1 isomer in comparison to S2 suggests more stability and lower reactivity (Table 1).

In addition to μ and η, the global electrophilicity index (ω), defined by Parr et al. [45] (Equation (5)), measures the energetic stabilization when the system acquires an additional electronic charge from the surrounding. The high ω value for the S2 isomer makes it more susceptible to accept electrons (Table 1).

The global reactivity descriptors cannot distinguish between the reactivities of different sites of a catalyst. On the other hand, since catalysis is mainly a local process a such distinction is important. Therefore, the present work shall use local reactivity descriptors as a way of identifying the most reactive sites of different clusters. Thereby, structural motifs will be identified that are expected to occur for clusters of other sizes and/or stoichiometries, also implying that the relative stability of the systems considered here is of secondary importance.

**Figure 2:**
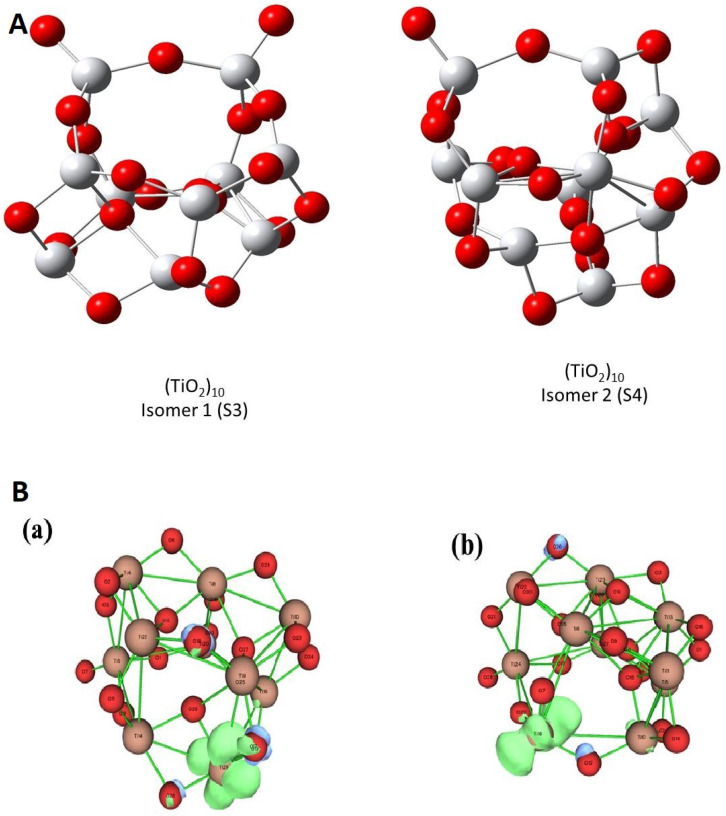
(**A**) Optimized structures of two isomers of (TiO${}_{2}$)${}_{10}$ labelled as S3 and S4. (**B**) Fukui functions ($f_{k}^{+}$) of S3 (**a**) and S4 isomers (**b**) of (TiO${}_{2}$)${}_{10}$ cluster in gas phase. Red spheres represent oxygen and grey spheres represent titanium atoms, respectively.

The active sites within a cluster have been identified by utilizing LRDs, i.e., first of all the Condensed Fukui Functions (CFFs). Table 2 contains the CFF values for isomers S1 and S2 of the Ti${}_{6}$O${}_{12}$ cluster in the gas phase. The more negative values of $f_{k}^{+}$ entails more propensity to accept an electron and the corresponding sites are prone to nucleophilic attacks. For the S1 isomer, Ti atoms are the sites susceptible to a nucleophilic attack. The O${}_{t}$ and under-coordinated Ti atoms in the S2 isomer are the sites prone to nucleophilic attacks. In order to check the influence of size on the reactive sites of the cluster, larger clusters (TiO${}_{2}$)${}_{10}$ (shown in Figure 2A) were considered. The condensed Fukui function plots shown in Figure 2B confirm that irrespective of size, terminal oxygens are the reactive sites of the cluster. Therefore, from now on, this study will concentrate on the Ti${}_{6}$O${}_{12}$ clusters.

Based on these descriptors, the possible mechanistic routes for CO oxidation using the Ti${}_{6}$O${}_{12}$ clusters were investigated in detail considering the different mechanisms mentioned above. Three pathways, labeled as I, II, and III, were studied to understand the mechanism of the CO oxidation. In pathways I and II either CO or O${}_{2}$ is initially adsorbed on the cluster whereas the other reactant remains in the environment until it reacts with the adsorbed species. These two possibilities are examined in pathway I and II, respectively. Here, the role of the catalyst (i.e., cluster) is determined by its binding of CO (in pathway I) or O${}_{2}$ (in pathway II). For pathway III, we considered co-adsorption of CO and O${}_{2}$.

Which pathway will be followed depends on the environmental conditions under which the experiment is performed. In particular, it depends on the temperature (T) and chemical potentials of O${}_{2}$, CO, and CO${}_{2}$. The thermodynamic stability is determined by the changes in its free energy, ΔG, as compared to pristine cluster and the non-interacting CO and O${}_{2}$ molecules. The change in the relative free energy (ΔG) at finite temperature and in an environment of CO, O${}_{2}$, and CO${}_{2}$ can be written as

(8) $$\;\;\;\;\;\;\;\;\;\;\;\;\;\;\;\;\;\;\;\;\;\;\;\;\;\;\;\;\;\;\Delta G\left(T\right)=G_{\mathrm{product}}-G_{\mathrm{cluster}}-\Delta \mu_{\mathrm{gas}}\left(T\right).$$

The last term describes the changes when the CO, O${}_{2}$, or CO${}_{2}$ molecules leave or enter the environment [46]. In the present study, however this term shall be ignored.

### 3.1. Pathway I: Addition of CO Followed by O${}_{2}$

To follow the pathway I, Ti was chosen as the site (more susceptible to nucleophilic attack with more negative *f*${}_{k}^{+}$) for the reaction for both S1 and S2 isomers. CO was placed near the reacting site and O${}_{2}$ was added to the environment. The pathway I proceeds through these two steps:

$$\begin{array}{cc} •\; & \mathrm{Step}1:\mathrm{Adsorption}\mathrm{of}\mathrm{CO}\mathrm{on}{\mathrm{Ti}}_{6}O_{12}\mathrm{cluster} \end{array}$$

(9) $$\begin{array}{cc} \; & \;{(\mathrm{TiO}}_{2}{)}_{6}+\mathrm{CO}\to {(\mathrm{TiO}}_{2}{)}_{6}-\mathrm{CO}\\ •\; & \mathrm{Step}2:\mathrm{Reaction}\mathrm{of}O_{2}\mathrm{with}\mathrm{pre}-\mathrm{adsorbed}\mathrm{CO} \end{array}$$

(10) $$\begin{array}{cc} \; & \;{(\mathrm{TiO}}_{2}{)}_{6}-\mathrm{CO}+O_{2}\to {(\mathrm{TiO}}_{2}{)}_{6}-O+{\mathrm{CO}}_{2}. \end{array}$$

**Figure 3:**
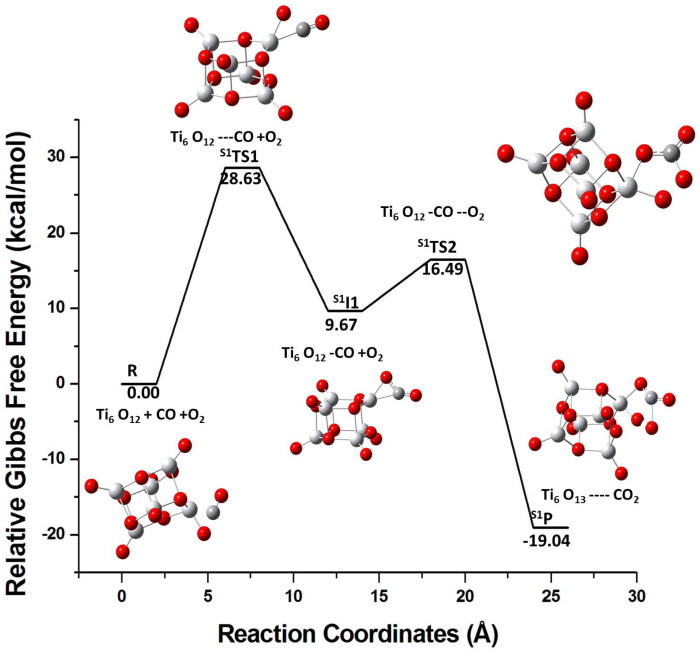
The relative free energy profile for the reaction of the S1 isomer with CO followed by a reaction with the O${}_{2}$ molecule. *R*, ${}^{S1}$TS1, ${}^{S1}$I1, ${}^{S1}$TS2, and ${}^{S1}$P represent reactants, transition state 1, intermediate state 1, transition state 2, and product, respectively.

The addition of CO to the S1 isomer leads to the ${}^{S1}$TS1 transition state where the (TiO${}_{2}$)${}_{6}$-CO complex (${}^{S1}$I1) is formed by passing an energy barrier of 28.63 kcal/mol. The C-O bond is elongated from 1.12 to 1.19 Å. Here, the lone pair of electrons in the CO molecule makes it a good nucleophile that binds at the electrophilic site of the cluster (notice, the Ti atoms in S1 have more negative $f_{k}^{+}$ values; see Table 2). Subsequently, the O${}_{2}$ molecule reacts with ${}^{S1}$I1 (step 2) and forms a stable complex ${}^{S1}$P (Product) by passing through the ${}^{S1}$TS2 state, that has a carbonate group attached to the Ti atom of the S1 cluster. The relative free energy profile is depicted in Figure 3.

**Figure 4:**
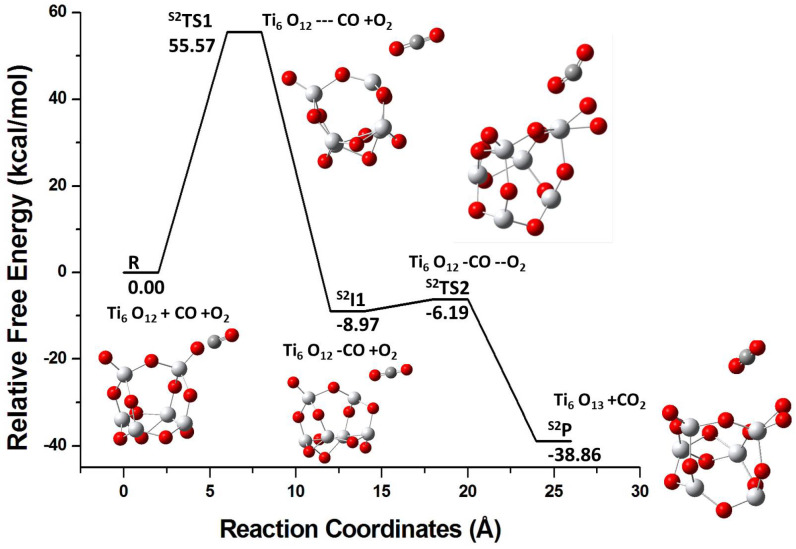
The energy profile for the reaction of the S2 isomer through pathway I R, ${}^{S2}$TS1, ${}^{S2}$I1, ${}^{S2}$TS2, and ${}^{S2}$P represent reactants (R), transition state 1 (TS1), intermediate state 1 (I1), transition state 2 (TS2), and product (P), respectively.

Figure 4 shows the energy profile for the same pathway I but for the S2 isomer. The CO molecule binds initially to the O${}_{t}$ atom of the cluster (step 1) via ${}^{S2}$TS1 and ultimately releasing 8.97 kcal/mol. The Ti-O${}_{t}$ bond elongates from 1.60 Å to 2.26 Å. The terminal oxygen atoms in the S2 isomer are electrophilic in nature (i.e., have more negative $f_{k}^{+}$ values; cf. Table 2). This explains the binding of the nucleophilic CO molecule to O${}_{t}$ in the S2 isomer. In step 2, as O${}_{2}$ is added to the ${}^{S2}$I1 intermediate state, CO${}_{2}$ detaches from the cluster and additionally 38.86 kcal/mol energy is released. Although CO binds to both S1 and S2, the addition of O${}_{2}$ to the pre-adsorbed CO complex leads to the release of CO${}_{2}$ only from the S2 isomer. The terminal oxygen atom in the S2 isomer has a strong electrophilic character, and its strong binding with CO facilitates the CO${}_{2}$ formation via the largely barrier-less TS. This observation is in agreement with the predictions that can be made on the basis of the GRD values. The S2 isomer has higher μ and lower η values that entail its greater reactivity in comparison to the S1 isomer (cf. Table 1). The higher ω value of the S2 isomer suggests that its reaction with CO imparts it higher stability (${}^{S2}$I1 is stabilized by 8.97 kcal/mol in Figure 4) than that observed for the S1 isomer (${}^{S1}$I1 is 9.67 kcal/mol higher in energy than the pristine cluster in Figure 3). These observations demonstrate that the geometric arrangement of the atoms in a cluster has strong influence on its reactivity.

**Figure 5:**
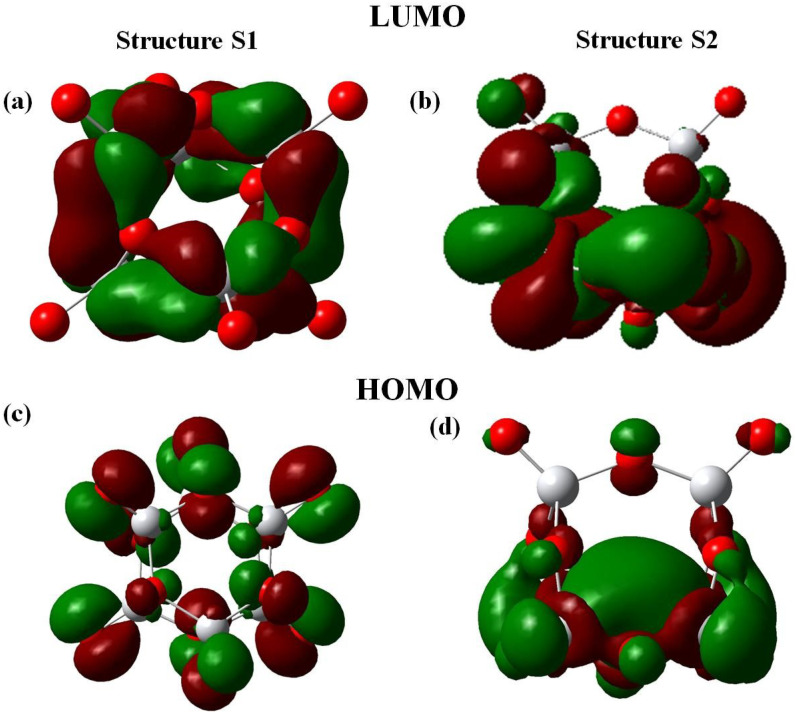
The frontier molecular orbitals of the clusters S1 and S2.

The difference in the reactivity of the two forms of Ti${}_{6}$O${}_{12}$ towards CO oxidation through pathway I will be further interpreted by analyzing the frontier orbitals. It is widely accepted that an electron donor (CO in the present case) binds more strongly at that site where the lowest unoccupied molecular orbital (LUMO) protrudes. For the binding to an electron acceptor (O${}_{2}$ in this case), the highest occupied molecular orbital (HOMO) is involved [47]. For the S1 isomer, CO is pre-adsorbed through a Ti-CO linkage as LUMO is localised on Ti atom (Figure 5a). For S2 the CO molecule binds to an O${}_{t}$ atom which is a electrophillic site (from FF values). It is to be noted that the barrier height in case of ${}^{S2}$TS1 is larger than that of ${}^{S1}$TS1. This may be due to differences in the spatial distrubution of the orbitals since the LUMO in case of S2 cluster is not completely localised on the terminal oxygen (binding site of CO) Figure 5b).

**Figure 6:**
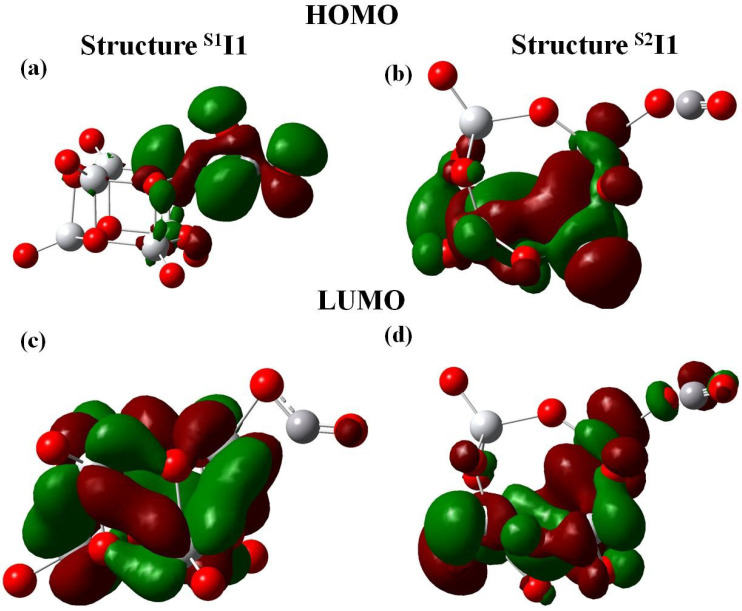
The frontier molecular orbitals of the intermediate states obtained in pathway I, ${}^{S1}$I1 and ${}^{S2}$I1.

This study has further considered the formation of CO${}_{2}$ and the carbonate complex in the presence of either the S2 or the S1 isomer by analyzing the HOMO and LUMO of the intermediates (${}^{S1}$I1 and ${}^{S2}$I1) as shown in Figure 6. For step 2 of the oxidation, the HOMO is localized to the carbon atom of CO in case of ${}^{S1}$I1 (Figure 6a). However, for ${}^{S2}$I1 the HOMO is localized to Ti atoms (Figure 6b). This difference can explain the formation of carbonate in the case of cluster S1 and the release of CO${}_{2}$ in the case of S2. The reactivity trend as obtained in the present study is well in tune with the conceptual DFT descriptors and reinforces the fact that geometry influences reactivity. Thus, both the GRD and the frontier orbitals help in rationalizing the reactivity of the two structural isomers of the Ti${}_{6}$O${}_{12}$ cluster in the CO oxidation reaction. The authors are convinced that these results are generally valid, in particular also for other TiO${}_{2}$ clusters for the CO oxidation reaction.

### 3.2. Pathway II: Addition of O${}_{2}$ Followed by CO

The pathway II explores another possibility of the sequential addition in which the O${}_{2}$ molecule is pre-adsorbed followed by the reaction with CO,

$$\begin{array}{cc} •\; & \mathrm{Step}3:\mathrm{Preadsorption}\mathrm{of}O_{2} \end{array}$$

(11) $$\begin{array}{cc} \; & \;{(\mathrm{TiO}}_{2}{)}_{6}+O_{2}\to {(\mathrm{TiO}}_{2}{)}_{6}-O_{2}\\ •\; & \mathrm{Step}4:\mathrm{Addition}\mathrm{of}\mathrm{CO} \end{array}$$

(12) $$\begin{array}{cc} \; & \;{(\mathrm{TiO}}_{2}{)}_{6}-O_{2}+\mathrm{CO}\to {(\mathrm{TiO}}_{2}{)}_{6}-O+{\mathrm{CO}}_{2} \end{array}$$

**Figure 7:**
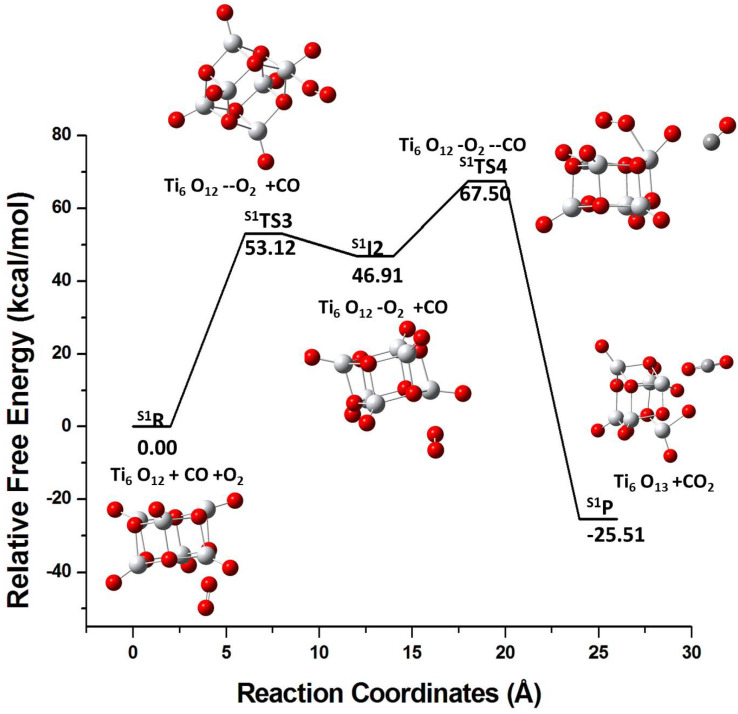
Energy profile for the reaction of the S1 isomer with O${}_{2}$ followed by the reaction with the CO molecule. R, ${}^{S1}$TS3, ${}^{S1}$I2, ${}^{S1}$TS4, and ${}^{S1}$P represents reactants, transition state 3, intermediate state, transition state 4, and product, respectively.

**Figure 8:**
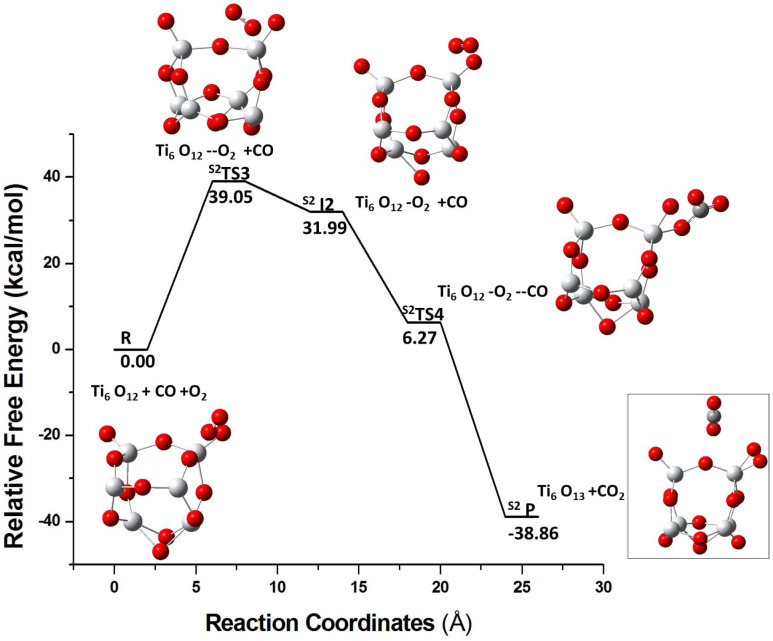
The energy profile for the reaction of S2 isomer with O${}_{2}$ followed by reaction with the CO molecule. R, ${}^{S2}$TS3, ${}^{S2}$I2, ${}^{S2}$TS4, and ${}^{S2}$P represents reactants, transition state 3, intermediate 2,transition state 4, and product, respectively.

The energy profile for pathway II is shown in Figure 7 for the S1 isomer. Initially, O${}_{2}$ is adsorbed on the cluster and the ${}^{S1}$I2 intermediate is formed via ${}^{S1}$TS3. After the addition of CO in step 2, CO${}_{2}$ and Ti${}_{6}$O${}_{13}$ are produced via ${}^{S1}$TS4 and 25.51 kcal/mol energy is released. The S2 isomer has a similar route for the pathway II (Figure 8). In pathway I, the pre-adsorbed CO molecule tends to bind to the electrophilic site of the cluster but the pre-adsorbed O${}_{2}$ does not bind strongly to the cluster. This can explain the same reactivity of the two isomeric forms in the pathway II since O${}_{2}$ shows no preference to bind to any cluster site in either form.

### 3.3. Pathway III: Co-Adsorption of CO and O${}_{2}$

**Figure 9:**
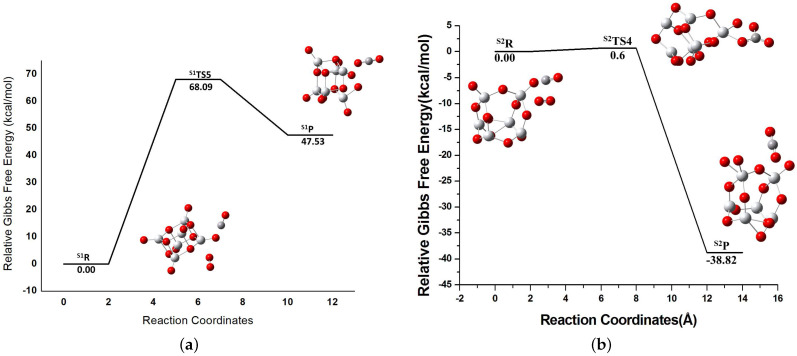
Energy profile for the LH mechanism for (**a**) the S1 isomer and (**b**) the S2 isomer. In the upper part, ${}^{S1}$R, ${}^{S1}$TS5, and ${}^{S1}$P represents reactant, transition state 5, and product for the reaction with the S1 isomer as catalyst. In the lower part, ${}^{S2}$R, ${}^{S2}$TS4, and ${}^{S2}$P represents reactant, transition state 4, and product for the reaction with the S2 isomer as catalyst.

The energy profile for both the isomers for the CO oxidation via this mechanism is depicted in Figure 9. It is based on the initial co-adsorption of CO and O${}_{2}$. Both S1 and S2 yield CO${}_{2}$ and Ti${}_{6}$O${}_{13}$ as the products. It is to be noted that the active oxygen which is bonded to CO to form CO${}_{2}$ is a terminal oxygen of the cluster and not part of the absorbed molecular oxygen. The reaction mechanism observed here is thus analogous to the MvK mechanism in which CO reacts with the oxygen of the catalyst. For the S1 isomer the TS barrier has a value of 68.09 kcal/mol (${}^{S1}$TS5 (Figure 9a)). On the contrary, in the case of the S2 isomer, the reaction is essentially barrierless. This may be due to the fact that the S2 isomer has a strong affinity towards CO because of its high reactivity as well as high electron affinity.

For all the three pathways, the molecular oxygen that is absorbed on the cluster is found to play an important role in activating the Ti-O terminal linkage.

### 3.4. Temperature Dependence

A complete understanding of the effects of temperature on the oxidation of CO in the presence of titanium oxide is crucial for practical applications.The effects of temperature have been included within a harmonic approximation and calculated the Gibbs free energies for reactant, transition state, and product. This requires the determination of the free energies of the clusters+gas molecules, and of the pristine clusters, as well as the chemical potentials of the gases, i.e., of CO, O${}_{2}$, and CO${}_{2}$. The present work follows a similar approach to that of Bhattacharya et al. [46].

For the reactant, i.e., isomer S1 of (TiO${}_{2}$)${}_{6}$ cluster in the presence of CO and O${}_{2}$ gases, the Gibbs free energy has been calculated as

(13) $$G_{R}=F_{\mathrm{Cluster}}+\mu_{O_{2}}+\mu_{CO},$$

for the transition state as

(14) $$G_{T}=F_{\mathrm{Cluster}+\mathrm{gas}},$$

and for the product as

(15) $$G_{P}=F_{\mathrm{Cluster}}+\mu_{{\mathrm{CO}}_{2}}.$$

For $G_{R}$ and $G_{P}$, the clusters and gas molecules are non-interacting. The free energies in all cases are calculated as below:

(16) $$F\left(T\right)=F^{\mathrm{translational}}+F^{\mathrm{rotational}}+F^{\mathrm{vibrational}}+F^{\mathrm{symmetry}}+F^{\mathrm{spin}}+E^{\mathrm{DFT}}$$

with

$$\begin{array}{cc} \; & F^{\mathrm{translational}}=-\frac{3}{2}k_{B}T\;\ln\left[\frac{2\pi mk_{B}T}{h^{2}}\right]\\ \; & F^{\mathrm{rotational}}=-k_{B}T\;\ln\left[8\pi^{2}{\left(\frac{2\pi k_{B}T}{h^{2}}\right)}^{3/2}\right]+\frac{1}{2}k_{B}T\;\ln\left(I_{A}I_{B}I_{C}\right)\\ \; & F^{\mathrm{vibrational}}=\sum_{i}\frac{h\nu_{i}}{2}+\sum_{i}k_{B}T\;\ln\left[1-\exp\left(-\frac{h\nu_{i}}{k_{B}T}\right)\right]\\ \; & F^{\mathrm{symmetry}}=k_{B}T\;\ln\;\sigma \\ \; & F^{\mathrm{spin}}=-k_{B}T\;\ln\;M. \end{array}$$

In Equation (16), $E^{DFT}$ is the total energy from a DFT calculation, *m* is the cluster mass, $I_{A,B,C}$ are the three moments of inertia of the cluster, $\nu_{i}$ is the *i*’th harmonic vibrational frequency, M is the spin multiplicity, and σ is a symmetry number.

**Figure 10:**
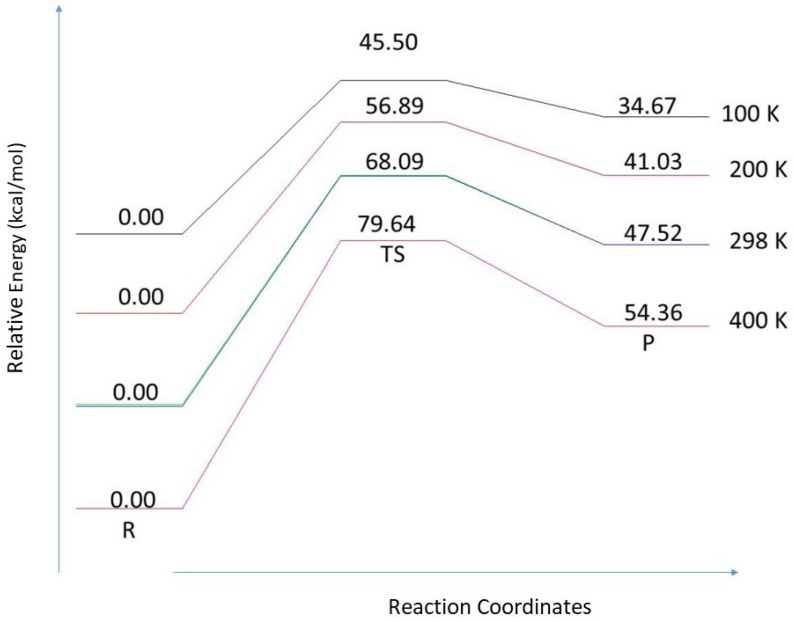
Effect of finite temperature on the relative Gibbs free energy for the reaction path 3 for isomer S1 of the (TiO${}_{2}$)${}_{6}$ cluster. Notice that the different curves have different zero-energy values.

The resulting relative Gibbs free energies were plotted for the reaction path 3 (the oxidation of CO in the presence of isomer S1 of (TiO${}_{2}$)${}_{6}$ cluster) in Figure 10 for different temperatures *T*. Raising *T* clearly increases the reaction barrier. Moreover, the reactants are stabilized compared to the product. This suggests that the oxidation of CO in the presence of S1 isomer of (TiO${}_{2}$)${}_{6}$ cluster shall be performed at lower *T*.

**Figure 11:**
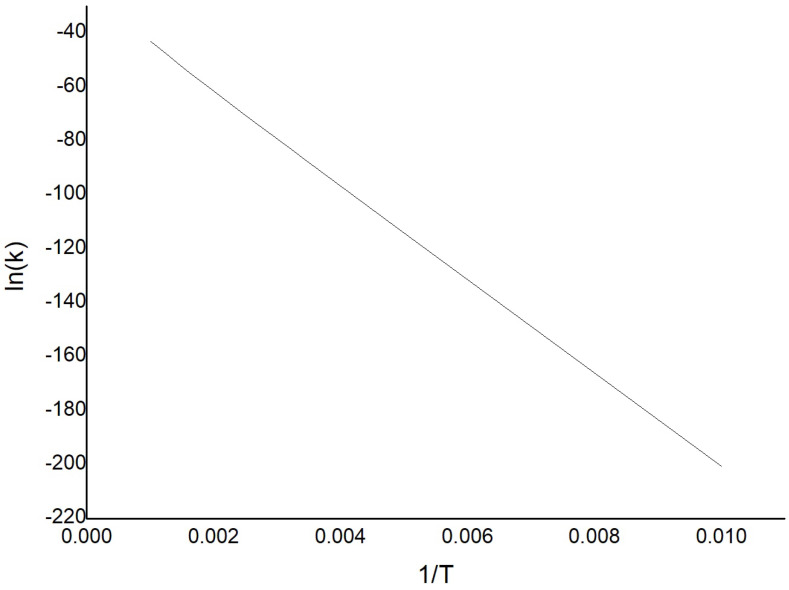
Plot of ln(k) vs 1/T for isomer S1 of the (TiO${}_{2}$)${}_{6}$ cluster.

Subsequently, the effect of temperature on the reaction was analysed by calculating the reaction rate constants *k* using the Eyring equation at different *T*. The plot of ln(k) vs 1/T is shown in Figure 11, where a behavior close to linear is observed. Thus, it can be assume that the Arrhenius equation is valid here. Then the intercept of ln(k) axis at 1/T=0, i.e., −26.21 s${}^{-1}$, is the value of ln(A), with *A* being the pre-exponential factor in the Arrhenius equation. Moreover, the slope of the curve equals then $-E_{a}/R$ from which the value for the activation energy, $E_{a}=34.77$ kcal/mol has been obtained.

## 4. Conclusions

In the present work, the effects of local geometry on the reactivity of TiO${}_{2}$ clusters for the CO oxidation using Ti${}_{6}$O${}_{12}$ were investigated for precise results. The authors are, however, convinced that the results are applicable to other sizes and/or stoichiometries of TiO${}_{2}$ nanoparticles as well.

Two structural isomers (S1 and S2) of Ti${}_{6}$O${}_{12}$ clusters were optimized using density functional calculations. The two isomers have contrasting symmetries, with the S1 isomer belonging to the high-symmetric D${}_{3d}$ point group while the S2 isomer has a distorted, low C${}_{s}$ symmetry.

Subsequently, their reactivity behavior was investigated whereby conceptual density functional descriptors were used to identify the reactive sites. The ER and LH mechanisms pertaining to sequential and simultaneous adsorption of CO and O${}_{2}$ on the (TiO${}_{2}$)${}_{6}$ clusters were studied. This study found that the reactivity of the cluster is strongly influenced by its local structure. The analysis of the activation barriers in the free-energy profiles leads to conclusion that the less symmetric S2 isomer is more efficient to catalyze CO oxidation.

Most importantly, the study could demonstrate that local and global reactivity descriptors were very helpful in predicting and rationalizing the catalytic activity of the systems under study. The authors believe that this is a general finding, whereby it should become possible to use those in identifying good catalysts.

The effect of raising the temperature on the oxidation of CO in the presence of S1 isomer of (TiO${}_{2}$)${}_{6}$ cluster was studied through the Gibbs free energies. The reaction barrier was found to become higher as *T* increases. The study calculated the reaction rate constants and using the slope of $\ln\left(k\right)-vs-\frac{1}{T}$ found the activation energy $E_{a}=34.77$ kcal/mol.

## Figures and Tables

**Table 1:** Global reactivity descriptors (GRDs) of the structural isomers S1 and S2 of Ti${}_{6}$O${}_{12}$ cluster in gas phase. All quantities are given in eV.

Cluster	VIE	VEA	η	μ	ω	E${}_{\mathrm{gap}}$
S1	10.98	5.08	5.90	−8.03	3.15	4.35
S2	8.42	4.90	3.52	−6.66	6.30	2.29

**Table 2:** The condensed Fukui functions ($f_{k}^{+}$ and $f_{k}^{-}$) of the atomic sites of S1 and S2 isomers of (TiO${}_{2}$)${}_{6}$ cluster (depicted in Figure 1) in gas phases.

Atom Label	Type of Atom (S1)	Charge (S1-Cluster)	$f_{k}^{-}$	$f_{k}^{+}$	Type of Atom (S2)	Charge (S2-Cluster)	$f_{k}^{-}$	$f_{k}^{+}$
1	Ti	1.460	−0.016	−0.069	Ti	1.367	−0.126	−0.040
2	O	−0.950	0.010	−0.023	Ti	1.369	−0.133	−0.299
3	O	−0.950	0.056	−0.023	O	−0.813	−0.039	−0.046
4	Ti	1.459	−0.022	−0.069	Ti	1.401	0.004	0.009
5	O	−0.509	0.121	0.121	O	−0.746	−0.044	−0.042
6	O	−0.510	0.102	0.120	O	−0.768	−0.045	−0.047
7	Ti	1.459	−0.017	−0.069	O${}_{t}$	−0.559	−0.090	−0.092
8	O	−0.950	−0.053	−0.023	O	−0.845	−0.057	−0.013
9	O	−0.950	0.056	−0.023	Ti	1.571	−0.062	−0.299
10	O	−0.950	0.053	−0.023	Ti	1.311	−0.134	−0.040
11	O	−0.509	0.121	0.121	Ti	1.403	0.001	0.017
12	Ti	1.459	−0.022	−0.069	O	−0.758	−0.044	−0.038
13	Ti	1.459	−0.017	−0.069	O	−0.757	−0.029	−0.069
14	O	−0.510	0.104	0.120	O${}_{t}$	−0.562	−0.086	−0.096
15	O	−0.950	0.010	−0.023	O	−0.449	−0.027	−0.018
16	Ti	1.459	−0.017	−0.069	O	−0.844	−0.030	−0.054
17	O	−0.510	0.104	0.120	O	−0.827	−0.026	−0.048
18	O	−0.510	0.102	0.120	O	−0.495	−0.033	−0.002

## Data Availability

Not applicable.

## References

[1] Yumura T., Amenomori T., Kagawa Y., Yoshizawa K. (2002). Mechanism for the formaldehyde to formic acid and the formic acid to carbon dioxide conversions mediated by an iron-oxo species. J. Phys. Chem. A.

[2] Lin H.Y., Chen Y.W., Wang W.J. (2005). Preparation of nanosized iron oxide and its application in low temperature CO oxidation. J. Nanoparticle Res..

[3] Li P., Miser D.E., Rabiei S., Yadav R.T., Hajaligol M.R. (2003). The removal of carbon monoxide by iron oxide nanoparticles. Appl. Catal. Environ..

[4] Wang Y.Z., Zhao Y.X., Gao C.G., Liu D.S. (2007). Preparation and catalytic performance of Co_3_O_4_ catalysts for low-temperature CO oxidation. Catal. Lett..

[5] Lopes I., Davidson A., Thomas C. (2007). Calibrated Co_3_O_4_ nanoparticles patterned in SBA-15 silicas: Accessibility and activity for CO oxidation. Catal. Commun..

[6] Stoyanova M., Konova P., Nikolov P., Naydenov A., Mehandjiev D., Christoskova S. (2006). Alumina-supported nickel oxide for ozone decomposition and catalytic ozonation of CO and VOCs. Chem. Eng. J..

[7] Weng Y.X., Du L.C., Zhang Q.L., Zhang L. (2005). A transient molecular probe for characterizing the surface properties of TiO_2_ nanoparticle in colloidal solution. Sci. Technol. Adv. Mater..

[8] Linsebigler A.L., Lu G., Yates J.T. (1995). Photocatalysis on TiO_2_ surfaces: Principles, mechanisms, and selected results. Chem. Rev..

[9] Grätzel M. (2001). Photoelectrochemical cells. Nature.

[10] Fujishima A., Honda K. (1972). Electrochemical photolysis of water at a semiconductor electrode. Nature.

[11] Buesser B., Grohn A., Pratsinis S.E. (2011). Sintering rate and mechanism of TiO_2_ nanoparticles by molecular dynamics. J. Phys. Chem. C.

[12] Hoffmann M.R., Martin S.T., Choi W., Bahnemann D.W. (1995). Environmental applications of semiconductor photocatalysis. Chem. Rev..

[13] Thompson T.L., Yates J.T. (2006). Surface science studies of the photoactivation of TiO_2_ new photochemical processes. Chem. Rev..

[14] Hagfeldt A., Boschloo G., Sun L., Kloo L., Pettersson H. (2010). Dye-sensitized solar cells. Chem. Rev..

[15] Widmann D., Behm R. (2014). Activation of molecular oxygen and the nature of the active oxygen species for CO oxidation on oxide supported Au catalysts. Accounts Chem. Res..

[16] Green I.X., Tang W., Neurock M., Yates J.T. (2013). Insights into catalytic oxidation at the Au/TiO_2_ dual perimeter sites. Accounts Chem. Res..

[17] Wang Y.G., Cantu D.C., Lee M.S., Li J., Glezakou V.A., Rousseau R. (2016). CO oxidation on Au/TiO_2_: Condition-dependent active sites and mechanistic pathways. J. Am. Chem. Soc..

[18] Fischer J.M., Hankel M., Searles D.J. (2015). Computational studies of the interaction of carbon dioxide with graphene-supported titanium dioxide. J. Phys. Chem. C.

[19] Vajda S., White M.G. (2015). Catalysis applications of size-selected cluster deposition. ACS Catal..

[20] Tyo E.C., Vajda S. (2015). Catalysis by clusters with precise numbers of atoms. Nat. Nanotechnol..

[21] Reddy B., Khanna S. (2004). Self-stimulated NO reduction and CO oxidation by iron oxide clusters. Phys. Rev. Lett..

[22] Himeno H., Miyajima K., Yasuike T., Mafuné F. (2011). Gas Phase Synthesis of Au Clusters Deposited on Titanium Oxide Clusters and Their Reactivity with CO Molecules. J. Phys. Chem. A.

[23] Nørskov J.K., Bligaard T., Logadottir A., Bahn S., Hansen L.B., Bollinger M., Bengaard H., Hammer B., Sljivancanin Z., Mavrikakis M. (2002). Universality in heterogeneous catalysis. J. Catal..

[24] Siu C.K., Reitmeier S., Balteanu I., Bondybey V., Beyer M. (2007). Catalyst poisoning in the conversion of CO and N_2_O to CO_2_ and N_2_ on Pt 4-in the gas phase. Eur. Phys. J. D.

[25] Schrader D., Schwarz H. (1995). CH and CC bond activation by bare transition-metal oxide cations in the gas phase. Angew Chem. Int..

[26] Boreskov G.K. (2003). Heterogeneous Catalysis.

[27] Doornkamp C., Ponec V. (2000). The universal character of the Mars and Van Krevelen mechanism. J. Mol. Catal. A Chem..

[28] Marom N., Kim M., Chelikowsky J.R. (2012). Structure selection based on high vertical electron affinity for TiO_2_ clusters. Phys. Rev. Lett..

[29] Arab A., Ziari F., Fazli M. (2016). Electronic structure and reactivity of (TiO_2_) n (n = 1–10) nano-clusters: Global and local hardness based DFT study. Comput. Mater. Sci..

[30] Bourcier S., Hoppilliard Y. (2002). B3LYP DFT molecular orbital approach, an efficient method to evaluate the thermochemical properties of MALDI matrices. Int. J. Mass Spectrom..

[31] Parr R.G., Pearson R.G. (1983). Absolute hardness: Companion parameter to absolute electronegativity. J. Am. Chem. Soc..

[32] Mulliken R.S. (1934). A new electroaffinity scale; together with data on valence states and on valence ionization potentials and electron affinities. J. Chem. Phys..

[33] Yang W., Parr R.G. (1985). Hardness, softness, and the fukui function in the electronic theory of metals and catalysis. Proc. Natl. Acad. Sci. USA.

[34] Glendening E.D., Badenhoop J.K., Reed A.E., Carpenter J.E., Bohmann J.A., Morales C.M., Landis C.R., Weinhold F. (2013). NBO 6.0: Natural bond orbital analysis program. J. Comput. Chem..

[35] Yang W., Mortier W.J. (1986). The use of global and local molecular parameters for the analysis of the gas-phase basicity of amines. J. Am. Chem. Soc..

[36] Seifert G., Porezag D., Frauenheim T. (1996). Calculations of molecules, clusters, and solids with a simplified LCAO-DFT-LDA scheme. Int. J. Quantum Chem..

[37] Aradi B., Hourahine B., Frauenheim T. (2007). DFTB+, a Sparse Matrix-Based Implementation of the DFTB Method. J. Phys. Chem. A.

[38] Becke A.D. (1993). Becke’s three parameter hybrid method using the LYP correlation functional. J. Chem. Phys..

[39] Lee C., Yang W., Parr R. (1988). Density-functional exchange-energy approximation with correct asymptotic behaviour. Phys. Rev. B.

[40] Rassolov V.A., Ratner M.A., Pople J.A., Redfern P.C., Curtiss L.A. (2001). 6-31G* basis set for third-row atoms. J. Comput. Chem..

[41] Bhattacharya S., Sonin B.H., Jumonville C.J., Ghiringhelli L.M., Marom N. (2015). Computational design of nanoclusters by property-based genetic algorithms: Tuning the electronic properties of (TiO_2_) n clusters. Phys. Rev. B.

[42] Schlegel H.B. (1982). Optimization of equilibrium geometries and transition structures. J. Comput. Chem..

[43] Legler C., Brown N., Dunbar R., Harness M., Nguyen K., Oyewole O., Collier W. (2015). Scaled quantum mechanical scale factors for vibrational calculations using alternate polarized and augmented basis sets with the B3LYP density functional calculation model. Spectrochim. Acta Part A Mol. Biomol. Spectrosc..

[44] Fukui K. (1981). The path of chemical reactions-the IRC approach. Accounts Chem. Res..

[45] Parr R.G., Szentpály L.v., Liu S. (1999). Electrophilicity index. J. Am. Chem. Soc..

[46] Bhattacharya S., Levchenko S.V., Ghiringhelli L.M., Scheffler M. (2014). Efficient ab initio schemes for finding thermodynamically stable and metastable atomic structures: Benchmark of cascade genetic algorithms. New J. Phys..

[47] Chretien S., Buratto S.K., Metiu H. (2007). Catalysis by very small Au clusters. Curr. Opin. Solid State Mater. Sci..

